# Immunomodulatory Treatment Impact on IVF Outcomes in KIR AA Genotype: Personalized Fertility Insights

**DOI:** 10.3390/medicina60060948

**Published:** 2024-06-06

**Authors:** Luana Seles, Ioana Alexandra Zaha, Mihai Luncan, Alin Bodog, Liliana Sachelarie, Mircea Sandor, Iulia Codruta Macovei, Erika Bimbo-Szuhai, Anca Huniadi

**Affiliations:** 1 Faculty of Medicine and Pharmacy, University of Oradea, 1st December Square 10, 410073 Oradea, Romania; luana.seles@yahoo.com (L.S.); drzahaioana@gmail.com (I.A.Z.); mishu_cx@yahoo.com (M.L.); drims75@yahoo.com (M.S.); icmek69@gmail.com (I.C.M.); bszera@gmail.com (E.B.-S.); ancahuniadi@gmail.com (A.H.); 2 Oradea County Hospital, Gheorghe Doja Street 65-67, 410169 Oradea, Romania; 3 Calla-Infertility Diagnostic and Treatment Center, Constantin A. Rosetti Street, 410103 Oradea, Romania; 4 Pelican Clinical Hospital, Corneliu Coposu Street 2, 410450 Oradea, Romania; 5 Department of Clinical Discipline, Apollonia University, 700511 Iasi, Romania

**Keywords:** implantation, immunological, KIR, fertilization, treatment, pregnancy

## Abstract

*Background and Objectives*: Recurrent implantation failure (RIF) affects 10% of couples undergoing in vitro fertilization (IVF), spurring exploration into tailored treatments to enhance implantation rates. Maternal immune tolerance towards embryos, particularly killer-cell immunoglobulin-like receptors (KIRs) on natural killer (NK) cells, is a focal point in RIF research. *Materials and Methods*: This retrospective cohort study, conducted at fertility clinic in Oradea, Romania, involved 65 infertile couples undergoing IVF treatment between January 2022 and December 2023. Couples were divided into two groups: KIR AA (Group A) and KIR Bx (Group B). *Results*: Factors such as age, type of infertility, oocytes retrieved, embryos produced, pregnancy rates in Group A without and with immunomodulatory treatment were documented. Group A, receiving immunomodulatory treatment, achieved a pregnancy rate of 47.8%, significantly higher than the 23.73% rate without treatment (*p* = 0.008). Group B had a higher mean patient age than Group A. However, miscarriage rates did not significantly differ between Group A with treatment and Group B (*p* = 0.2457), suggesting comparable outcomes with immunomodulation. *Conclusions*: The impact of immunological factors on recurrent implantation failure is being more and more emphasized and warrants the attention of specialists in human reproduction. Uterine natural killers and their function though KIR receptors deserve particular attention as immunomodulatory treatment may improve pregnancy rates in patients with KIR AA haplotype.

## 1. Introduction

Recurrent implantation failure (RIF) is a challenge encountered by 10% of couples undergoing in vitro fertilization (IVF) [1]. Promising strides have been made in improving implantation rates through personalized treatments aimed at addressing the root causes. In the realm of RIF research, a significant focus has been placed on immunological factors, particularly the maternal immune response to embryos. Defined as the failure to achieve a clinical pregnancy after transfer of at least four good-quality embryos in a minimum of three fresh or frozen cycles in women under the age of 40 years, RIF is a multifactorial condition with both embryonic and uterine factors playing crucial roles [1,2,3] and the revised definition from ESHRE (European Society of Human Reproduction and Endocrinology) latest good practice guide states that a minimum two good quality embryos have been transferred without obtaining a pregnancy [4].

Natural killer (NK) cells, with their killer-cell immunoglobulin-like receptors (KIRs), play a crucial role in the complex interactions between the maternal immune system and the developing fetus [2]. Specific KIR alleles (KIR AA) have garnered attention in RIF investigations due to the hypothesis that certain KIR-HLA-C combinations may contribute to implantation failures. Recent studies have underscored the potential of KIR genotyping as a predictive tool for RIF. The identification of these combinations holds the promise of tailored approaches in assisted reproductive technology, encompassing immunomodulatory interventions and customized embryo transfers [5,6]. 

Among the various factors implicated in RIF, immunological factors have garnered considerable interest, particularly the role of maternal immune tolerance towards the embryo [7].

Killer-cell immunoglobulin-like receptors (KIRs) constitute a cluster of surface proteins situated on natural killer (NK) cells, a pivotal component of the immune system involved in combatting viruses and certain cancers. Encoded by a gene family located in chromosome 19 in humans, KIRs are instrumental in regulating also immune responses, particularly within the maternal–fetal interface during pregnancy [8,9].

At this interface lies the interaction between maternal KIRs and fetal human leukocyte antigen (HLA) molecules expressed on trophoblast cells. The KIRs interact with specific HLA-C (human leukocytes antigen genotype C) ligands presented by the trophoblast, playing a vital role in placental development and immune tolerance during pregnancy [9,10]. This interplay profoundly influences maternal immune responses, impacting processes in trophoblast invasion and placental development [4,5,6,7,8,9].

Emerging evidence from multiple studies underscores the significance of specific combinations of maternal KIR genes and fetal HLA-C genes in either predisposing to or protecting against certain pregnancy-related complications, including preeclampsia and recurrent miscarriage. These findings underscore the pivotal role of KIRs in modulating maternal immune responses to ensure favorable pregnancy outcomes [5,6,7,8,9,10].

The polymorphic nature of KIR genes and their HLA ligands contributes to the diversity in immune responses among individuals, which can have profound implications in pregnancy outcomes [11].

KIR A and KIR B are the two primary classes into which KIR genes can be divided. Two of the seven genes that make up KIR A are activating genes [12]. In contrast, KIR B usually comprises two or more activating genes in addition to a variable number of genes. One notable mutation is found in Caucasians: around 80% of them have a mutation in their KIR genes. This mutation causes the surface of NK cells to lack activated KIR receptors in people with the KIR AA genotype [12,13,14]. 

The polygenicity of KIR haplotypes is due to contraction and expansion of the KIR locus and the KIR going through a non-allelic recombination [11]. The haplotypes are grouped into A and B according to their genes. The framework genes for KIR are KID3DL3, 72 KIR3DL2, KIR3DP1, and KIR2DL4 found in all individuals [12].

The inability to activate KIR receptors may have an effect on NK cell activity, which is crucial for identifying and eliminating contaminated cells. This genetic variant might affect vulnerability and immunological responses [13].

For instance, the presence of activating KIR alleles in combination with HLA-C2 ligands has been associated with increased rates of successful pregnancies, whereas the absence of these activating receptors has been linked to RIF [13].

The HLA-A, HLA-B, HLA-C, and HLA-G allotypes are the activating and inhibitory ligands. The KIR is shown in the maternal and paternal HLA-C allotypes. The two kinds of HLA-C, C1 and C2, differ based on whether asparagine or lysine is present on the α-domain. The inhibitory KIR2DL2 and KIR2DL3 are bound by HLA-C1, while KIR2DL1 and KIR2DS1 are bound by HLA-C2. The trophoblast invasion and placentation during pregnancy are determined by HLA-C exposure [14]. KIR has ends that are telomeric (tel A or B) and centromeric (cen A or B). KIR A haplotype consists of cen-A and tel-A, whereas the KIR B haplotype is defined by combinations of cen-A, cen-B with tel-A, tel-B. KIR2DL3, KIR2DL2, and KIR2DS2 are found in Cen-A, KIR3DL1 and KIR2DS4 are found in Cen-B, while KIR2DS1 and KIR3DS1 are found in Tel-A [15]. 

Furthermore, the role of KIR AA in RIF is not only limited to direct interactions with HLA ligands but also extends to the regulation of NK cell cytotoxicity and cytokine production. Abnormal NK cell activity, influenced by KIR-HLA interactions, has been proposed as a mechanism contributing to RIF [14]. 

Elevated levels of uterine NK cells and altered expression of KIR receptors have been observed in women with RIF, suggesting a dysregulation of the local immune environment conducive to implantation [15]. Both copies of haplotype A defines KIR AA; on the other hand, the presence of haplotype B defines KIR Bx (AB, BB). Therefore, KIR AA is A/A (0–1 activating KIR), A/B (1–6 activating KIR), or BB (3–10 activating KIR) [6].

KIR genes are on chromosome 19, and HLA genes are on chromosome 6; KIR on the NK cells bind to HLA class I [15,16,17]. Every pregnancy has a unique KIR and HLA-C combination from both maternal KIR and HLA class I variants and paternal HLA-C variants [18].

Overall, the observed elevated levels of uterine NK cells and altered expression of KIR receptors in women with RIF suggest a dysregulated local immune environment that is not conducive to successful implantation [14]. Understanding these immune dysfunctions may lead to targeted interventions aimed at improving implantation rates and reproductive outcomes in affected individuals.

Recent studies have also highlighted the potential of KIR genotyping as a predictive tool for RIF [9,10,11]. The identification of specific KIR-HLA-C combinations in women with a history of RIF could guide personalized approaches in ART, such as immunomodulatory treatments or tailored embryo transfer strategies. 

However, the clinical application of these findings is still in its infancy, and further research is required to establish robust protocols.

The implications of KIR AA in recurrent implantation failure represent a complex interplay of genetic, immunological, and environmental factors. 

In summary, KIRs expressed on NK cells serve as pivotal regulators of immune responses at the maternal-fetal interface, crucial for the establishment and maintenance of pregnancy. Comprehensive comprehension of the intricate interplay between maternal KIRs and fetal HLA molecules is indispensable for elucidating the mechanisms underlying pregnancy-related disorders and may offer insights into the development of diagnostic and therapeutic approaches in obstetrics and reproductive medicine [6,7,8].

Immunosuppressive treatments for RIF in IVF may involve various medications aimed at modulating the maternal immune response to improve implantation success. Some commonly used agents are corticosteroids orally or iv like prednisone, intravenous immunoglobulin, intralipid therapy, low-dose aspirin, low-molecular weight heparin, and progesterone. Along with these ones, there are others that have been investigated, such as tumor necrosis factor (TNF) inhibitors like etanercept, granulocyte colony-stimulating factors (G-CSF) like filgrastim, and selective immunotherapy that involves targeting T-lymphocytes like tacrolimus. In all cases, immunomodulatory treatments for patients undergoing IVF experiencing RIF are administered based on immunologists’ recommendations in select cases [9,10,11].

The objectives of the study are to demonstrate/research the immunological cause as a potential etiology of implantation failure and the effectiveness of the immunomodulatory treatment addressed to it in increasing the pregnancy rate.

## 2. Materials and Methods

This retrospective cohort study was carried out at a university-affiliated fertility clinic in Oradea, Romania, involving 65 infertile couples who underwent IVF treatment between January 2022 and December 2023. The study was approval by the Ethics Committee under the reference number 870/15 December 2020 and was conducted at the Calla—Infertility Diagnostic and Treatment Center. It was recorded at Clinical Trials.gov with trial number NCT06264206.

**Figure 1:**
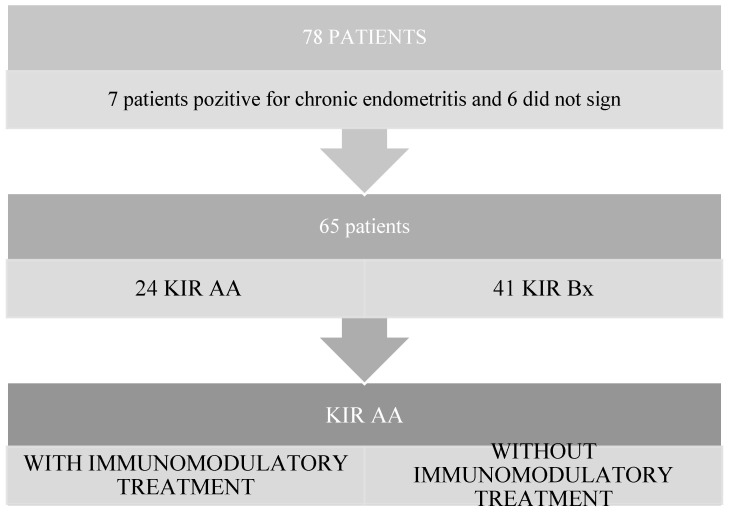
Workflow.

The study included 65 patients who were divided according to haplotype. Group A—KIR AA included a number of 24 patients who had at least one embryo transfer without immunomodulatory treatment followed by an embryo transfer with immunomodulatory treatment. Group B—KIR Bx included a number of 41 patients with the haplotype who did not receive immunomodulatory treatment as illustrated in Figure 1.

Fertility screening in all patients included clinical examination, hormonal testing (including HOMA, prolactin, and thyroid), sperm count, pelvic ultrasound, and thrombophilia testing. Patients with recurrent implantation failure (RIF) had at least two failed embryo transfers with good quality embryos. 

Inclusion criteria comprised infertility diagnosis, IVF procedure with two top-quality embryos, negative chronic endometritis test, and normal hysteroscopic findings. 

Exclusion criteria included absence of signed consent; refusal of hysteroscopy; uterine abnormalities; thin endometrium; endometrial polyps; pelvic cancer; acute inflammatory disease; Asherman syndrome; chronic endometritis; thrombophilia, genetic or acquired; dysregulated thyroid pathology; and oocyte or spermatozoa donor. 

KIR genotyping is a genetic test that allows the maternal KIR gene repertoire of a patient to be determined. Through a PCR-SSO analysis of 16 KIR genes (2DL1, 2DL2, 2DL3, 2DL4, 2DL5, 2DS1, 2DS2, 2DS3, 2DS4, 2DS5, 3DL1, 3DL2, 3DL3, 3DS1, 2DP1 and 3DP1) utilizing DNA extracted from a blood sample.

The infertility diagnosis based on the WHO (World Health Organization) definition is any couple that does not achieve pregnancy after 12 months of regular unprotected sexual intercourse. The ESHRE (European Society of Human Reproduction and Embryology) definition of infertility states that it is a disease characterized by the failure to establish a clinical pregnancy after 12 months of regular unprotected sexual intercourse or due to an impermeant of a person’s capacity to reproduce either as an individual or with their partner.

The 65 patients consisted of group A, which was patients with KIR AA, and group B, which included patients with KIR Bx. 

Group A included 24 patients that had at least 2 embryo transfers in the past with good-quality embryos and did not become pregnant or patients that had a history of recurrent pregnancy loss and an IVF indication and had been tested for the KIR genotype. 

Group B included 41 patients that had an IVF indication and at least 2 previous embryo transfers with no pregnancy or patients that had a history of recurrent pregnancy loss and an IVF indication and had been tested for the KIR genotype. 

Assessing the effect of immunomodulatory therapy on KIR AA patients’ pregnancy rates is the main goal. Comparing the conception rates of KIR Bx and KIR AA groups with and without therapy is the secondary outcome.

### 2.1. Embryo Transfer Preparation

In both groups, the patient’s evaluation was carried out within the first 3 days of their menstrual cycle, involving a transvaginal ultrasound evaluation and hormonal balance evaluation. Afterwards, the treatment with either estradiol 2 mg three times per day or monitorization of ovulation started. After 7 days, the ultrasound evaluation was carried out for the patients that were on estradiol preparation protocol, and the endometrial thickness was monitored so we could increase the dosage. On day 15 of preparation, if the endometrial thickness was over 7 mm, progesterone was added, and on the 5th day, the embryo transfer performed. If there was a natural cycle, the ultrasound evaluation was carried out on day 12–14; at the peak of LH, we added progesterone; and on the 5th day, the embryo transfer was performed. The dose of progesterone was 1200 mg intravaginally (UTROGESTAN^®^ 200 mg, Besins Healthcare, (London, UK) Ltd. Medicines). On the day of embryo transfer, the progesterone level needed to be >10.6 ng/mL. For group A (KIR AA) after an immunological consult, treatment with Prednisone^®^ 15 mg daily (SINTOFARM) and Tacrolimus^®^ 2 mg daily (Astellas Ireland Co., Ltd., Killorglin, County Kerry, Ireland) started 3–5 days before the embryo transfer and was carried out until the indication of the immunology specialist. Also, after immunological counseling, KIR AA patients received Intralipid iv perfusion on the day of ET.

Embryo transfer was performed under transabdominal ultrasound guidance using Cook Medical’s Guardia™ Access Nano. After 10 days, a beta HCG (human chorionic gonadotropin) test was conducted using maternal serum, and medication was continued. A transvaginal ultrasound was scheduled for 4 weeks after embryo transfer to confirm clinical pregnancy.

### 2.2. Statistical Analysis 

Descriptive statistical analysis was performed using SPSS Statistics SPSS 26.0 (IBM SPSS Software) for Windows. ANOVA and sample *t*-tests were used to determine immunomodulatory treatment impact.

## 3. Results

### 3.1. Baseline Characteristics of Groups

The mean age of the patients was 35.25 for group A and 36.6 for group B, with no statistical difference between the two groups (*p* = 0.2818). The mean number of oocytes retrieved was 11.12 for group A and 10.95 for group B, with no statistical difference in our study; see Table 1. In both group A and group B, the majority were urban patients.

From a statistical point of view, there are no significant differences between the two groups (*p* > 0.001). However, it is evident that Group B tended to have a higher average age value than Group A, with a mean age difference of −1.36, as shown in Table 1.

### 3.2. Descriptive Statistics Group A and Group B

Descriptive statistics for group A are described in Table 2.

In group A we have obtained a pregnancy rate of 9.375% without immunomodulatory treatment, afterwards we have obtained a pregnancy rate of 39.78% with immunomodulatory treatment this being a significant statistical difference *p* < 0.001 (Table 3).

The descriptive parameters of the patients in group A are the same; they are in fact the same patients. What differs is the pregnancy rate, which was higher in these patients after receiving the immunomodulatory treatment; see Table 3. After the immunosuppressive treatment, the pregnancy rate became comparable to that of the B group.

In group B, we obtained a pregnancy rate of 34.99%, which is higher than in group A without treatment (9.375%) and becomes comparable with treated group A (39.28).

### 3.3. Comparation of Parameters for Group A and Group B 

From a statistical point of view, there are no significant differences between the two groups (*p* > 0.001). However, it is evident that Group B tends to have higher average values for homa, fertilized oocytes, and no. of ETs compared to Group A, with a negative mean age difference, as shown in Table 4.

There are significant differences between the two groups (*p* < 0.001) in blast rate and the number of ETs, Table 4.

There are various statistical findings and trends between Group A and Group B, particularly concerning age differences, treatment effects within Group A, and differences in several parameters between the two groups.

## 4. Discussion

In the paper “ESHRE good practice recommendation of recurrent implantation failure”, if RIF is suspected in the couple, there is a recommended follow-up consisting of re-assessment of lifestyle factors for both partner re-assessment of endometrial thickness, assessment of antiphospholipidic antibodies and antiphospholipidic antibodys syndrome in case of risk factors. The paper also states practitioners can consider testing the karyotype for both partners 3D ultrasound, hysteroscopy, endometrial function testing, chronic endometritis testing, assessment thyroid function, and progesterone levels in the late follicular and mid-luteal phase. The same paper states that we should not conduct vitamin D testing, microbiome profiling, peripheral or uterine NK cell testing, uterine T lipocyte testing, assessment of blood cytokine levels, assessment of HLA-c compatibility, or assessment of mitochondrial DNA content or sperm DNA fragmentation [18]. 

Still, the notion that an excessive maternal immune response to the implanting embryo has an unfavorable effect is widely accepted. The ESHRE recommendation reflects the lack of consensus between the studies regarding the reliable methods of assessing the distribution of uterine natural killer and the degree in which the number of uterine natural killer reflects their function in the endometrium. Functional tests including the constitution of receptors—KIRs may have more clinical value, as Woon et al. state in their study. Also, these uterine natural killers from the endometrium could act like a biosensor and their inadequate activation might truly be a cause of RIF [19,20]. 

During early pregnancy development, the involvement of placentation, particularly the decidua and spiral arteries, is well recognized, with key components being the interactions between KIR and trophoblast HLA-C [16].

Within the IVF population, recurrent implantation failure (RIF) occurs in approximately 10% of cases. From the most important factors in RIF, we underline maternal age, BMI, tobacco use, and embryonic euploidy, and all of these would make a fair 50–70% of causes [21,22].

In this study, the general characteristics of the two groups KIR AA and KIR Bx are similar, with a slight difference in the mean value of the age in female patients. Group B has a slightly higher mean value of age compared to group A, but not significantly statistic. According to our study the patients that have been diagnosed with KIR AA and had immunomodulatory treatment with prednisone and tacrolimus had a higher pregnancy rate compared to the same patients that did not have any treatment before (*p* = 0.008). 

Immunosuppressive treatments for RIF in IVF may involve various medications aimed at modulating the maternal immune response to improve implantation success. Some commonly used agents are corticosteroids, orally or IV, like prednisone; intravenous immunoglobulin; intralipid therapy; low dose aspirin; low molecular weight heparin; and progesterone. 

There are studies that have presented the benefits of prednisone on the uterine environment and showed the impact on the receptivity. Prednisone or prednisolone are often prescribed to suppress immune activity and reduce inflammation, potentially creating a more favorable environment for implantation [23,24,25,26,27,28].

Sun et al. compared 2 groups, one having 10 mg prednisone as treatment and one that received a placebo treatment, and in this study, the pregnancy rate was similar between groups, and apparently the biochemical pregnancy rate was higher in the prednisone group without a higher pregnancy rate [29,30]. Additionally, the use of tacrolimus in RIF patients has been emphasized since 2014 [28,29,30,31]. This was the first trial using tacrolimus in recurrent implantation failure as an adjuvant therapy. Having these results, our study promotes the use of prednisone and tacrolimus in targeted patients during frozen embryo transfer protocols for the impact and improvement of pregnancy rate overall and is one of the first studies that use immunomodulatory treatment in same group of patients that have not received the treatment before with a pregnancy rate of 39.58% [28].

In our study, the patients taking the treatment with tacrolimus had a higher pregnancy rate compared to the pregnancy rate in the same patients before the treatment. Tacrolimus suppresses Th1 activity and enhances the expression of IL-10 and LIF. The increase of Th1 causes miscarriage and implantation failure. The effect of tacrolimus is beneficial on implantation by targeting this mechanism [25].

Using tacrolimus in RIF has been highlighted since 2014, comparing patients that received the treatment with tacrolimus over a period of 16 days total before and after the embryo transfer with those who did not. The group that had the treatment had a pregnancy rate of 64% compared to the placebo group [32].

Treatment approaches have been proposed for patients with elevated uterine natural killers or evidence of inadequate KIR function receptors consisting of lipid infusions and glucocorticoid administration but until adequate randomized control trials will be published on a great number of patients there recommendation in ESHRE guidelines is still the one stated before. 

Also, the influence of KIR haplotype on reproductive outcomes has been studied by other scientific groups such as Maftei et al., with results that indicate that the miscarriage rate was significantly lower and live birth rate significantly higher in the Bx haplotype [33]. 

Regarding patients with KIR AA and oocyte donation, the live birth rate was reduced compared to KIR Bx patients, even with oocyte donation. This disparity could potentially be mitigated by selecting C1 HLA-C donors in KIR AA patients. However, oocyte donation was not included in our study due to concerns about exacerbating immune responses [34].

Another important aspect is HOMA values, group B with the KIR Bx genotype demonstrates higher values, not correlating with increased fertilized oocytes and subsequent embryo transfers. Elevated HOMA values are linked to reduced ovarian sensitivity, necessitating higher doses of gonadotropin in obese patients undergoing IVF [20]. Furthermore, insulin resistance is associated with a heightened risk of miscarriage, emphasizing the importance of addressing this issue prior to IVF, particularly in PCOS patients [35,36,37,38].

We recognize that our study has several strengths and limitations. To our knowledge, this is one of the fewest studies to elucidate the incidence of immunological RIF and the efficiency of immunomodulatory treatment (prednisone plus tacrolimus) for improving the prognosis of immunological RIF. The identification and validation of such treatment holds promise in reproductive health, providing a more nuanced understanding of immune factors and contributing to clinic assessments. An additional strength of our study is the inclusion of an adequate control group, the same patients who previously had an embryo transfer without immunomodulatory treatment and afterwards having the ET in the same conditions but with the treatment. Despite these strengths, we recognize that our study has also limitations: the small number of couples registered (in the future, a larger cohort of patients will be needed); the lack of multicentric validation; the lack of information about the embryo euploidy; and the fact that KIR haplotypes have a gene variation according to the geographic region.

The current study opens up new possibilities for RIF diagnostic and treatment approaches while highlighting the critical role that KIR-HLA interactions play in implantation success. However, it is difficult to get firm results in RIF research due to the diversity of study methodologies, populations, and diagnostic criteria.

In order to clarify the precise function of Kir AA in RIF and to apply these findings to therapeutic practice, it is therefore essential that future research concentrate on bigger cohorts, uniform definitions, and longitudinal designs.

## 5. Conclusions

The impact of immunological factors on recurrent implantation failure is being more and more emphasized and warrants the attention of specialists in human reproduction. Uterine natural killers and their function though KIR receptors deserve particular attention as immunomodulatory treatment may improve pregnancy rates in patients with KIR AA haplotype. 

Immune cells play a crucial role in pregnancy development. However, the specific significance of the KIR HLA-C complex remains unclear, necessitating further research to determine the relevance of immunomodulatory treatments in patients with the KIR AA genotype undergoing IVF and experiencing recurrent pregnancy loss.

## Figures and Tables

**Table 1:** Characteristics of groups.

Age	N	Mean	SD	Mean_A-B_	t	*p* Value
Group A	24	35.25	5.1659	−1.36	1.0856	0.2818
Group B	41	36.609	4.5685			
**Environment**	**N**	**Urban**	**Rural**	**Mean**	**SD**	***p* Value**
Group A	24	18	6	12	6	0.021
Group B	41	37	4	20.5	16.5	0.067
**Number of Oocytes**	**N**	**Mean**	**SD**	**Mean_A-B_**	**t**	***p* Value**
Group A	24	11.125	7.1839	0.17	0.1091	0.9134
Group B	41	10.95	5.3645			

**Table 2:** Descriptive statistics group A and group B.

Characteristics—Group A	Mean	SD
HOMA index	3.586	2.674
Fertilized oocytes	7.75	5.4
Blastocyst	3.54	3.08
Blastocyst rate (%)	47.60	23.36
No. pregnancy with immunomodulatory treatment	0.41	0.49
No. pregnancies without immunomodulatory treatment	0.166	0.372
Pregnancy rate (%) with immunomodulatory treatment	39.58	47.82
Pregnancy rate (%) without immunomodulatory treatment	9.375	23.73
No. ETs with immunomodulatory treatment	0.958	0.538
No. ETs without immunomodulatory treatment	1.458	0.9565
**Characteristics—Group B**	**Mean**	**SD**
HOMA index	2.4036	0.748
Fertilized oocytes	8.41	4.378
Blastocyst	3.243	1.693
Blastocyst rate (%)	40.20	10.538
No. oocytes	10.95	5.364
Pregnancy rate (%)	34.99	35.9859
No ETs	2.658	1.117

**Table 3:** Pregnancy rate with and without immunomodulatory treatment in group A.

Characteristics—Group A	Mean	SD	Mean_A-B_	t	*p* Value
Pregnancy rate (%) with immunomodulatory treatment	39.28	47.82	0.25	1.94	0.008
Pregnancy rate (%) without immunomodulatory treatment	9.375	23.73			
No. ETs with immunomodulatory treatment	0.958	0.538	0.50	2.1846	0.0041
No. ETs without immunomodulatory treatment	1.458	0.9565			

**Table 4:** Comparation of parameters for Group A and Group B.

Characteristics	Mean_A-B_	SED	t	*p* Value
HOMA index	−1.824	1.858	1.858	0.9821
Fertilized oocytes	−0.66	1.253	0.5306	0.5976
Blastocyst	0.30	0.602	0.4947	0.6225
Blastocyst rate (%)	7.401	4.303	1.7201	0.0090
No. ETs	−1.70	0.247	6.883	0.0001

## Data Availability

The original contributions presented in the study are included in the article, further inquiries can be directed to the corresponding author.
